# Novel frameless LINAC radiosurgery solution for uveal melanoma

**DOI:** 10.3389/fonc.2024.1365197

**Published:** 2024-03-25

**Authors:** Louis Cappelli, Mehak Khan, Sudheshna Vemula, Christina Hum, Haisong Liu, Yan Yu, Yingxuan Chen, Yechi Zhang, Muhammad Sharif, Wenyin Shi

**Affiliations:** ^1^ Department of Radiation Oncology, Thomas Jefferson University, Philadelphia, PA, United States; ^2^ Sidney Kimmel Medical College, Thomas Jefferson University, Philadelphia, PA, United States; ^3^ Department Mechanical Engineering, University of New York, City College, New York, NY, United States

**Keywords:** uveal melanoma, radiosurgery, radiation treatment, 3D printing, noninvasive, novel treatment

## Abstract

**Introduction:**

Radiation treatment has replaced enucleation as an organ-preservation treatment for patients with uveal melanoma (UM). We developed a novel non-invasive, frameless LINAC based solution for fractionated stereotactic radiosurgery (fSRS) treatment.

**Methods:**

We designed and constructed the a stereotactic ocular localization box that can be attached and indexed to a stereotactic LINAC tabletop. It contains adjustable LED lights as a gaze focus point and CCD camera for monitoring of the patient’s eye position. The device also has 6 infrared spheres compatible with the ExacTRAC IGRT system. Treatment plans were developed using iPLAN Dose version 4.5, with conformal dynamic arcs and 6MV photon beam in flattening filter free mode, dosed to 50Gy in 5 fractions. During treatment, patients were instructed to stare at the light when a radiation beam is prepared and ready for delivery. Eye movement was tracked throughout treatment. Residual setup errors were recorded for evaluation.

**Results:**

The stereotactic ocular localization box was 3D-printed with polylactic acid material and attached to the stereotactic LINAC tabletop. 10 patients were treated to evaluate the feasibility, tolerability and setup accuracy. Median treatment time for each arc is 17.3 ± 2.4 seconds (range: 13.8-23.4). After ExacTRAC setup, the residual setup errors are -0.1 ± 0.3 mm laterally, -0.1 ± 0.3 mm longitudinally, and 0 ± 0.2 mm vertically. The residue rotational errors are -0.1 ± 0.3 degree pitch, 0.1 ± 0.2 degree roll, and 0 ± 0.2 degree couch rotation. All patients received treatment successfully.

**Conclusion:**

We successfully developed a novel non-invasive frameless mask-based LINAC solution for SRS for uveal melanoma, or other ocular tumors. It is well tolerated with high set up accuracy. Future directions for this localization box would include a multi-center trial to assess the efficacy and reproducibility in the fabrication and execution of such a solution for UM therapy.

## Introduction

Uveal melanoma (UM), while a rare tumor, is the most common intraocular malignancy found in adults (1). Of all melanoma cases reported in the United States, 5% are UM (2), with most arising in the choroid and fewest in the iris (3). UM is commonly seen among older demographics, ages 50 to 70 (2, 4). Metastases that result from UM are nonresponsive to immune checkpoint inhibitors. Therefore, UM that has metastasized, particularly to the liver, has a poor prognosis and is fatal within one year (4, 5). Prognosis decreases with increasing metastases to the liver, lungs, skin, and bones; older age at diagnosis; extraocular extension; large tumor margins; secondary glaucoma; or a large tumor thickness (6).

Effective treatments for UM typically focus on preserving the eye and possibly maintaining some vision. The Collaborative Ocular Melanoma Study (COMS), a major randomized trial in the U.S., found no survival advantage of enucleation over iodine plaque-125 radiotherapy (7). Plaque radiotherapy is a viable option for eligible patients, offering benefits like eye preservation, pain relief, and in some cases, a chance for vision enhancement (8).

Radioactive episcleral eye-plaque brachytherapy is the most well-established treatment modality utilized in the management of UM. Other forms of radiation treatment may be considered too, such as stereotactic radiotherapy (SRT) and stereotactic radiosurgery (SRS) with Gamma Knife or LINAC, or proton beam radiotherapy (7). Proton beam radiotherapy has emerged as an effective treatment for patients with UM. Proton therapy has the physical qualities of sharp dose fall off and uniform dose distribution that allow for minimal dose to near-by critical structures. In certain clinical scenarios involving larger tumors, recurrent disease, and tumors located closer to the optic nerve, proton therapy is the preferred treatment modality. However, proton beam therapy currently has limited access for many patients. There are currently less than 40 proton therapy centers in the United States, and many do not offer eye treatment. Treatments remain costly and limited due to the extensive infrastructure and support required to maintain such facilities (8).

Gamma Knife and LINAC based SRS/SRT have also been demonstrated to be effective treatment options in the management of UM (6, 9–12). However, the availability of Gamma Knife and LINAC is much higher than that of proton therapy. The current clinical solution requires treatments to use a frame-based approach with the need for retrobulbar anesthesia and/or the surgical fixation of the eye in order to be effective (13–16). In order for such treatments to be maximally safe and effective, it is imperative to have accurate treatment setup of the patient and alignment to the UM target (17, 18). As a result, very few facilities are able to offer such treatment. A patient-friendly solution is required to overcome the logistical challenges and the necessity for anesthesia, which is not suitable for everyone. This solution should be frameless, eliminating the need for procedures like retrobulbar anesthesia and surgical fixation of the eye (16, 19, 20).

The goal of this study is to develop a novel LINAC based frameless SRS solution for patients with uveal melanoma and other ocular tumors, that is easily adaptable to all existing stereotactic LINAC equipment. Specifically, a novel ocular localization box was developed using 3D-printing technique. Ten patients with uveal melanoma treated at our institution were included in this study to evaluate the feasibility of this novel 3D-printed localization box for clinical application.

## Methods

### Ocular localization box design and fabrication

We designed a novel ocular stereotactic localization box and fabricated it with an Ultimaker-S5 3D-printer. Specifically, the 3D-printed localization box can be attached and indexed to the LINAC treatment couch table, which has six degrees of freedom. The localization box was able to accommodate treatment necessities: an LED light and a charge-coupled device (CCD) camera (Logitech C920 Hd Pro Webcam). The LED light permitted a gaze focus point for the patient throughout the duration of the therapy, while a CCD camera allowed for real-time monitoring of the patient’s eye position.

The thermoplastic immobilization mask was modified to expose the eyes, allowing therapists to monitor the motion of the patient’s eye during treatment. During the treatment, instructions are given to the patient to gaze or perform a “stare-hold” as needed to ensure accurate and safe radiation treatment delivery.

The box was designed with both upper-layer and lower-layer brackets. The upper-layer bracket hosted the LED light for patient gaze focus and the CCD camera (Logitech C920 HD Pro Webcam) for real-time monitoring of patient eye position. The lower-layer bracket was secured to the LINAC couch table. Adjustment of the localization box is imperative for appropriate LINAC-SRS and SRT treatment, and indexable translation of the localization box was permitted in both the lateral and longitudinal directions.

### Feasibility evaluation

With Institutional Board Review approval, 10 patients with uveal melanoma were treated with fractionated SRS using our in-house ocular stereotactic localization box. Treatment planning was carried out with Brain Lab iPLAN (BrainLab, Munich, Germany). Radiation treatment was delivered with TrueBeam STx (Varian, Palo Alto, CA) using high definition multileaf collimator (HD-MLC) and ExacTRAC (BrainLab, Munich, Germany) on board daily imaging guidance. All patients were fitted with custom-made BrainLab thermal plastic masks for immobilization, with modifications to expose the eyes. Patients underwent CT simulation with ocular localization box. Patients were instructed to gaze at the gaze focus point during CT. Treatment planning MRI and CT images were obtained and fused. All patients had thin cut (1-1.5 mm) axial fat suppressed post-contrast and T2 MRI. The gross tumor volume (GTV) was defined as tumor on T1 post-contrast and T2 thin cut MRI. GTV was reviewed and may be modified by the ophthalmologists based on retina examination report. There is no CTV expansion. The planning target volume (PTV) was the GTV with 2 mm margin. Critical normal structures such as optic nerves, chiasm, lens, and brainstem were also contoured. The radiation planning used dynamic conformal arcs. The patients were treated with fractionated SRS (fSRS) to a dose of 50 Gy in 10 Gy fractions, every other day. ExacTRAC setup results with ocular localization box were evaluated.

## Results

**Figure 1:**
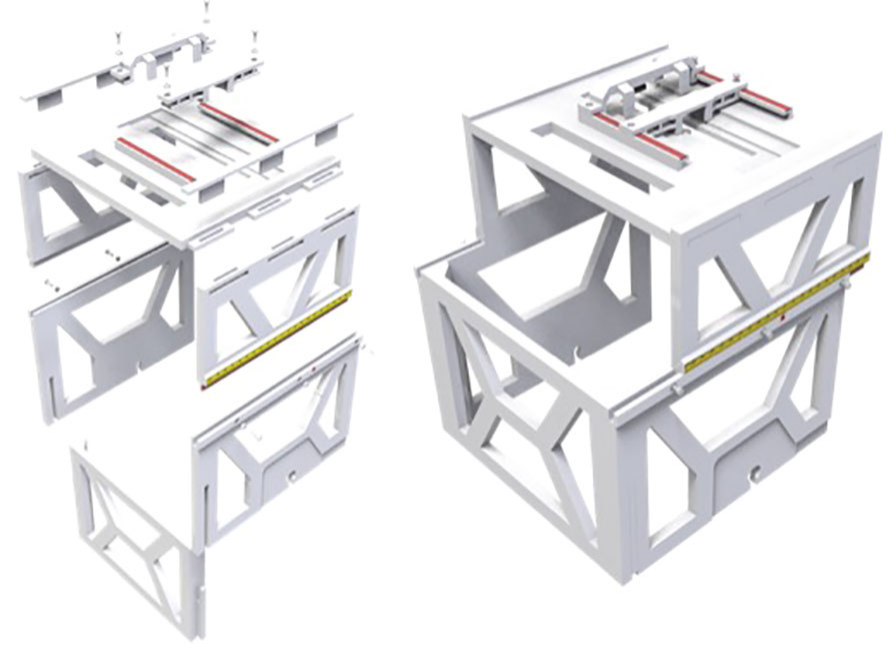
Design and fabrication of the 3D-printed localization box.

**Figure 2:**
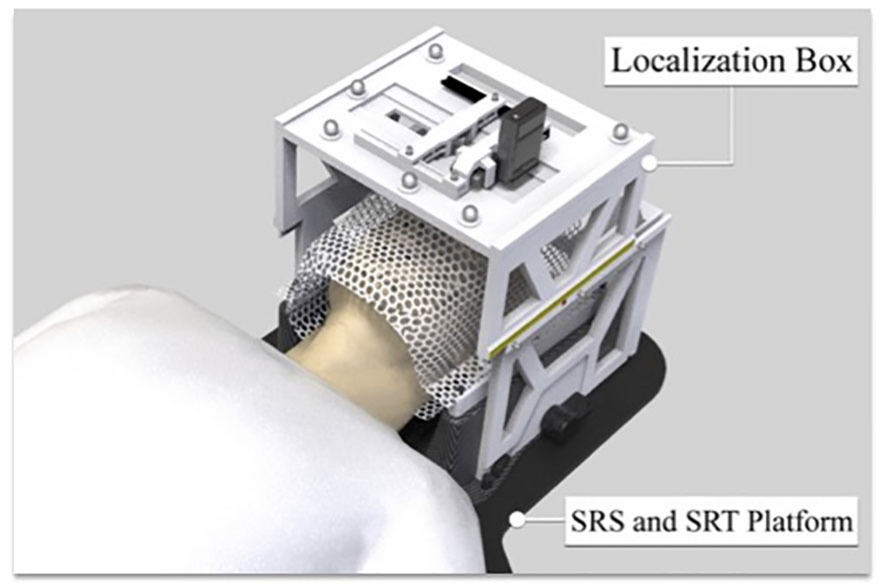
Localization box compatible with BrainLab frameless SRS and SRT system.

We developed a patient friendly, low cost, non-invasive, frameless LINAC SRS solution for ocular tumor, such as uveal melanoma. We designed the ocular localization box with the following goals: 1. Compatible with stereotactic thermoplastic mask system, and can be indexed to the LINAC table top, 2. Compatible with image guidance system, such as ExacTRAC system, 3. Has a patient gaze focus point, with adjustable position for left or right eye, 4. Has a CCD camera for real time monitoring of patient eye position, with adjustable position for left or right eye. 5. Fabricated with material that does not significantly attenuate radiation beam energy. The ocular localization box was 3D printed (**Figure 1**) with polylactic acid (PLA) material, which has an electron density of 1.06 and an attenuation factor of 0.95. The frame of the localization box has a base and upper layer bracket. The position of the upper layer bracket is adjustable. A total of 6 BrainLab infrared refraction markers are mounted on top of the upper layer bracket. The localization box is attachable to the stereotactic LINAC tabletop and can be locked and indexed (**Figure 2**).

**Figure 3:**
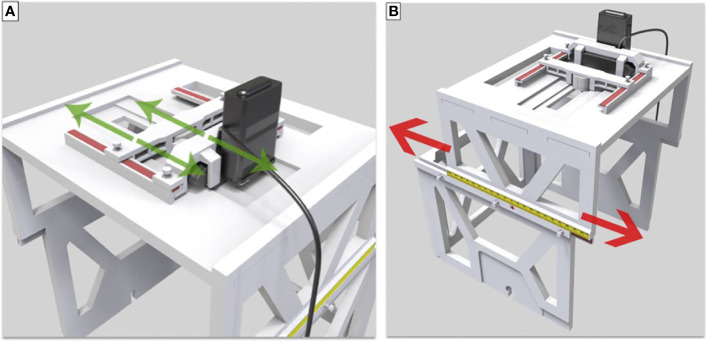
**(A)** Translation and lock of LED and camera in the lateral direction. **(B)** Movement and lock in the longitudinal direction.

The design and fabrication of one 3D-printed localization box was completed, and the localization box is compatible with current LINAC-SRS and SRT BrainLab software. The addition of the localization box allowed for successful flexible positioning of the camera and LED lights in both lateral and longitudinal directions (**Figure 3**). The LED lights allowed for a gaze focus point for the patient undergoing SRS or SRT, and the CCD camera allowed for real-time monitoring of the eye position for uveal melanoma treatment.

The upper-layer bracket and small plastic screws locking in the box allowed for the successful placement of the LED lights and the CCD camera and lateral movement along the convex edge. The slide groove and convex rail positioned between the upper-layer and lower-layer brackets secured with small plastic screws enabled appropriate longitudinal movement. The scale on the upper-layer bracket was used to denote the unique position of each patient during SRS or SRT therapy for uveal melanoma, making this localization box a universal tool for all patients with individual setup.

**Figure 4:**
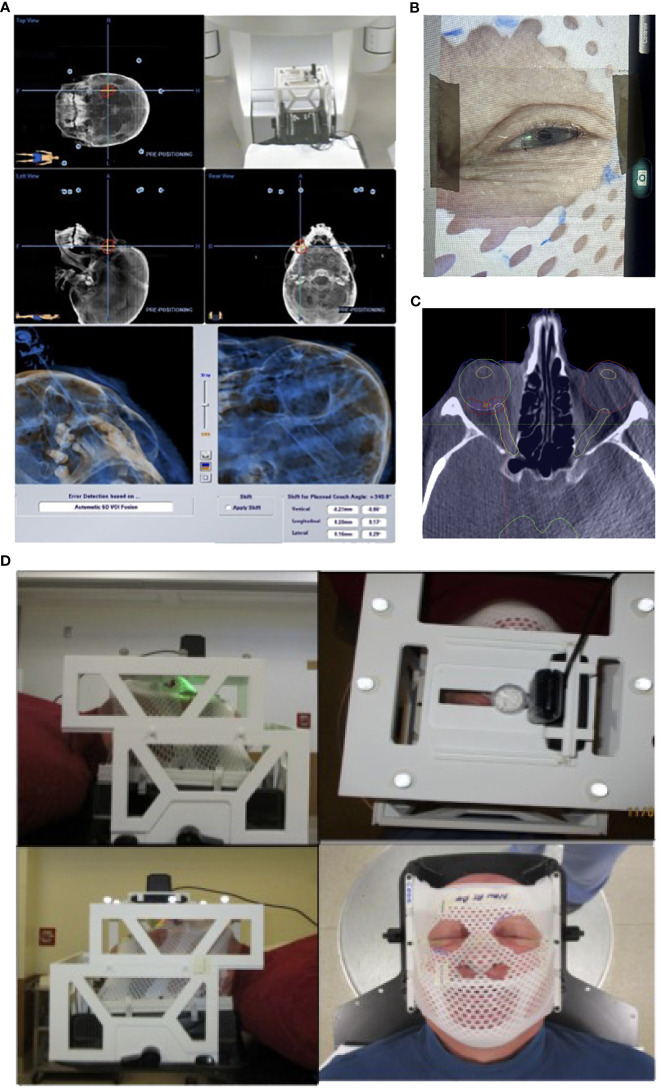
Patient setup and treatment workflow illustration. **(A)** Initial patient setup with ExacTRAC. **(B)** Therapist will instruct patient “stare-hold” while acquiring CBCT at treatment position. **(C)** Physician will review CBCT images, with focus on evaluating the treatment side optic nerve position compared to that of planning CT image. **(D)** Therapist will instruct patient to “stare-hold” during the radiation beam delivery for each arc, and instruct patient to relax during arc transitions.

Importantly, the localization box was fully compatible with the BrainLab platform for patient and treatment setup started with initial alignment via ExacTRAC imaging and CBCT. Through the use of gaze localization, patients were then instructed by a therapist to perform a “stare-hold” while acquiring CBCT at the time of treatment position, which was to be reviewed by a physician. Patients were instructed to relax their eyes after CBCT. The patient may need to repeat the “stare hold” simulation several times until a comfortable and reproducible position of the pupil is achieved. Patient setup and alignment were evaluated by the physician for accuracy to the target, with additional focus on the treatment side position of the optic nerve compared to that of the planning CT scan. Following approval, the therapist would again instruct the patient to perform the “stare-hold” during radiation beam delivery for each treatment arc, allowing the patient to relax during arc transitions. (**Figures 4A–D**)

**Figure 5:**
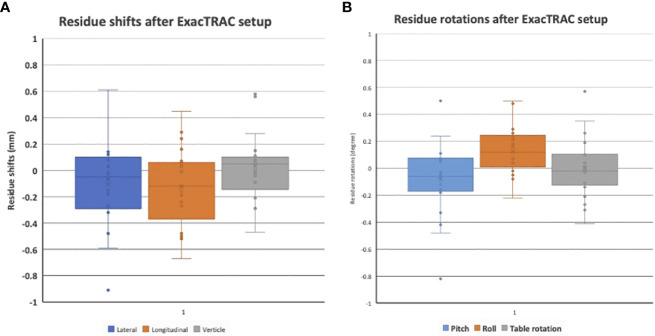
**(A)** Residual setup in shift errors following setup with ExacTRAC. **(B)** Residual setup errors in rotation following setup with ExacTRAC.

For the feasibility evaluation, 10 uveal melanoma patients were treated using the localization box. All the patients were treated with dynamic arc plans with 4-5 couch positions and arcs. FFF (flattening filter free) mode, with a dose rate of 1400 cGy/min was used. As a result, each arc delivery time was 10-15 seconds. As a result, no patient had trouble keeping the gaze for the delivery of radiation. ExacTRAC was successfully used for initial set up and intrafraction verification for all patients at all couch angles. Residual shifts, or the remaining rotational and translational shifts calculated by the ExacTRAC system following patient repositioning was measured. The ExacTRAC data showed that all patients achieved within 1 mm translational shift and 1 degree of rotational setup accuracy throughout the treatment course, which is under the pre-set tolerance (**Figure 5**). All patients were able to receive and finish treatment successfully.

## Discussion

Eye and potential vision preservation strategy is the treatment of choice of patients with uveal melanoma. Radioactive episcleral eye-plaque brachytherapy is the most well-established treatment modality utilized the management of UM. However, technological advances in radiation therapy treatment techniques have permitted the use of SRT as an effective eye preserving treatment modality for therapeutic management of uveal melanomas. Dieckman et al. demonstrated that hypofractionated treatments like LINAC-SRS/SRT have feasible and comparable local tumor control and toxicity profiles in a 4-year review of 90 patients (1). As a result, improvements to the efficacy and accuracy of LINAC-SRT/SRS became of interest, but the limitations and burdens of such practices requiring retrobulbar anesthesia and/or fixation of the eye are significant and limit access of patients to such treatment. This led to the development of a non-invasive approach to SRS/SRT for UM. In this study, we developed and investigated the feasibility of a novel non-invasive frameless LINAC based SRS/SRT solution for ocular tumors.

The localization box was designed with attention to reproducibility, design flexibility, and compatibility to existing infrastructure. Importantly, the device described here may significantly lower the overall cost of treatment compared to more traditional SRT treatment options. Primarily, the use of a frameless SRT eliminates the operational costs associated with patient immobilization in other forms of SRT, or with the use of SRS as traditionally used in this treatment setting. Previously, suction devices and anesthetic blocking had been used to stabilize the eye during uveal melanoma treatment (2). Such invasive measures can be risky, expensive and time consuming for a patient. In contrast, the localization box is a non-invasive measure that reduces risk of surgical complications and enhances patient comfort. We developed the device using readily available polylactic acid (PLA) filament and 3D-printing due to increasing accessibility to this technology in medical facilities. As a result, the production process can be streamlined with rapid prototyping of the device at the institution where the patient receives treatment. Additionally, 3D-printing enables the creation of adjustable parts that fit the patient’s unique anatomy, reducing the need for costly future modifications. These factors contribute to increased user-friendliness of the 3D-printed stereotactic localization box.

Treatment of uveal melanoma can constitute the use of several radiotherapeutics. They include eye-plaque brachytherapy, proton beam radiotherapy, and SRT/SRS. While each therapeutic offers its own unique advantages and limitations, the choice of treatment modality depends on the size of the tumor, stage of cancer, overall patient health, and accessibility (3). Brachytherapy is the most commonly used technique, but use is restricted to small or medium size lesions and has potential side effects of radio-induced retinopathy or neovascular glaucoma (4). Proton beam therapy is a form of external radiation using protons to target tumors in or near sensitive areas, however, it has typically higher operations costs, limiting widescale use and availability (5).

In contrast, SRT and SRS have a benefit in that they do not require preliminary surgery for tumor localization. Instead, 3D imaging techniques, including CT and MRI, are used during treatment to define the tumor borders. Notably, there are several variants of SRT, such as gamma knife and cyber-knife that have been employed in the medical setting. The Cyberknife method enables irradiation of malignant tissue with a high level of precision and few adverse effects by adapting the irradiation target in conjunction to the patient’s movement (3). Gamma knife is a single session, high dose radiation with effective local tumor control (6). However, the use of retrobulbar anesthesia in these invasive frame-based techniques poses significant challenges regarding patient comfort, safety, procedural workflow, and frame stability. While other modes of patient immobilization have been explored, including conjunctival surgery with muscle sutures, suction attachment, and extraocular muscle sutures, they pose the same disadvantages (7, 8).

Previous research has elucidated the critical role of ocular fixation and precision in treatment delivery within the realm of proton therapy. Foti et al. highlighted the innovative use of MRI imaging to identify the positions of tantalum clips, which are sutured to the sclera, serving as markers to delineate tumor boundaries accurately. This crucial information about the location of tantalum seeds is integral not only for formulating the treatment plan but also for real-time monitoring of the patient’s eye position during therapy, ensuring the precise delivery of radiotherapy (21). Similarly, Cirrone et al. shared insights from the CATANA proton therapy facility, detailing their patient immobilization strategies that also utilize tantalum clips as reference points throughout the planning and treatment stages. They describe a process where the patient, immobilized using a thermoplastic mask and bite block, focuses on a light source. Subsequently, two orthogonal X-rays are captured to verify the eye’s position. Utilizing EYEPLAN software, the team compares these images with simulated reconstructions to achieve optimal alignment (22). These studies underscore the sophistication and precision of current proton therapy techniques, emphasizing the importance of accurate ocular fixation in enhancing treatment efficacy.

There are also other non-invasive ocular fixation methods reported in the literature. Iskanderani et al. introduced a novel approach utilizing a customized head frame and vacuum pillow system, which significantly reduced patient movement without the need for invasive procedures. This method not only improved the reproducibility of treatment sessions but also enhanced patient tolerance and comfort during the procedure (23). In addition, Tsui et al. used a patented device consisting of a plastic frame, centered red LED light, and a camera on the contralateral side where the patient would gaze and focus at the red light through treatment. A paper ruler was attached to the periocular area to track the movement of the eye, with the eye positioning being monitored with the camera over the contralateral eye (24).

In our institution, the use of the novel localization box in conjunction with BrainLab and LINAC-SRS achieved a seamless 1 mm and 1 degree setup accuracy for treatment of uveal melanoma. Moreover, the localization box presents a simple and intuitive interface; with each arc time being only 10-15 seconds, patients reported little difficulty in holding effective gaze during the treatment duration. The main limitation of our device is the manual passible eye tracking performed by the therapist. This could lead to potential unwanted variability in treatment setup and delivery that could compromise the accuracy of the radiotherapy. Future innovation should be geared toward reducing this potential for error.

It should also be mentioned that the high dose rate selected for treatment of 1400 cGy/min was done to reduce treatment delivery time per arc. This could negatively impact the safety of treatment if the patient was unable to maintain “stare-hold” given the latency between the camera and therapist reaction time to discontinue treatment; however, after treating all these reported patients, we noticed that once a patient was coached to “stare-hold” and the pupil fell into the same position, it did not have any noticeable movement at all during the <30 seconds of treatment delivery time.

Our prototype of a 3D-printed stereotactic localization box offers promising prospects for LINAC-SRT in clinical practice. While the system demonstrated favorable outcomes in our experience, the size of our patient population was limited. Future studies should aim to expand the study size and compare and quantify the long-term clinical outcomes of toxicities of LINAC/SRT with the 3D localization box compared to other radiotherapeutics. A long-term goal of work is to improve the ability to target uveal melanoma with irregular borders and expand the use of the device in treatment of other ocular tumors. A successful fSRS system can provide an affordable and conservative treatment option, improving both patient comfort and safety.

## Conclusion

A novel ocular localization box was created with 3D-printing to use in conjunction with the already existing BrainLab platform for LINAC-SRS for uveal melanoma, or other ocular tumors. Future direction for this localization box would include a multi-center trial to assess the efficacy and reproducibility in the fabrication and execution of such solution for UM therapy.

## Data availability statement

The raw data supporting the conclusions of this article will be made available by the authors, without undue reservation.

## Ethics statement

The studies involving humans were approved by Thomas Jefferson University Institutional Review Board. The studies were conducted in accordance with the local legislation and institutional requirements. Written informed consent for participation was not required from the participants or the participants’ legal guardians/next of kin because the study is retrospective and did not enroll human participants. The study is based on technical and data review of the treatment process.

## Author contributions

LC: Validation, Visualization, Writing – original draft, Writing – review & editing. MK: Writing – original draft. SV: Writing – original draft. CH: Data curation, Project administration, Writing – original draft. HL: Conceptualization, Data curation, Formal analysis, Investigation, Methodology, Project administration, Software, Supervision, Validation, Visualization, Writing – original draft, Writing – review & editing. YY: Conceptualization, Data curation, Formal analysis, Investigation, Methodology, Project administration, Software, Writing – original draft. YC: Conceptualization, Data curation, Formal analysis, Investigation, Methodology, Project administration, Software, Writing – original draft. YZ: Conceptualization, Data curation, Investigation, Methodology, Writing – original draft. MS: Writing – original draft. WS: Conceptualization, Data curation, Formal analysis, Investigation, Methodology, Project administration, Software, Supervision, Validation, Visualization, Writing – original draft, Writing – review & editing.
